# Copy number determination of sperm-borne small RNAs implied in the intergenerational inheritance of metabolic syndromes

**DOI:** 10.1261/rna.080480.125

**Published:** 2025-08

**Authors:** Lisa König, Victoria Guggenberger, Kristeli Eleftheriou, Zsuzsanna Pinter, Alessandro Marotto, Christoph R. Kreutz, Mark Wossidlo, Virginie Marchand, Yuri Motorin, Matthias R. Schaefer

**Affiliations:** 1 Division of Cell and Developmental Biology, Center for Anatomy and Cell Biology, The Medical University of Vienna, A-1090 Vienna, Austria; 2 Institute of Organic Chemistry, Center for Molecular Biosciences (CMBI), University of Innsbruck, A-6020 Innsbruck, Austria; 3 Université de Lorraine, SMP IBSLor, EpiRNA-Seq Core facility and IMoPA, UMR 7365 CNRS-UL, 54000 Nancy, France

**Keywords:** copy numbers, intergenerational inheritance of acquired traits, spermatocyte, tRNA fragments

## Abstract

Mammalian spermatocytes harbor small RNAs that are mostly degradation products of abundant noncoding RNAs, including ribosomal RNA-derived small RNAs (rsRNAs) and tRNA-derived RNAs (tDRs). Notably, tDRs have been implicated in inheriting paternally acquired traits in rodents. Direct experimental proof for this notion comes from manipulating fertilized murine oocytes through microinjection of small RNA preparations, resulting in metabolic changes measurable in the offspring. How these paternally transmitted small RNAs could function mechanistically in the developing zygote remains to be understood. Since nothing is known about how many small RNAs are required for functional impact, we aimed to determine the copy numbers of specific small RNAs contained in a single spermatocyte. Using hybridization-based methods that avoid amplification-induced biases, we estimated average copy numbers for specific tDRs and rsRNAs per murine spermatocyte. While the measured numbers allow an approximation of how many rRNA- and tRNA-derived RNAs enter a murine oocyte during fertilization, the magnitude of these numbers underscores the need for remaining cautious when interpreting the effects of nonphysiological copy numbers of small RNAs that were used to manipulate a biological system.

## INTRODUCTION

RNA sequencing revealed that mammalian sperm contain various classes of RNAs, which are potentially transferred into the oocyte upon fertilization (Krawetz et al. 2011; Peng et al. 2012; Das et al. 2013; Jodar et al. 2013; Sendler et al. 2013). Among these sperm-borne RNAs, degraded ribosomal RNAs (ribosomal RNA-derived small RNAs, rsRNAs) represented the most abundant fraction in humans, pigs, stallions, bulls, and mice (Johnson et al. 2011; Peng et al. 2012; Chen et al. 2016; Sharma et al. 2016; Shi et al. 2021). Mature spermatocytes also contain degradation products of transfer RNAs called tRNA-derived RNAs or tDRs (Peng et al. 2012; Holmes et al. 2023). Importantly, tDRs were implicated as active agents responsible for the inheritance of extra-chromosomal information between two generations in mice (Chen et al. 2016; Sharma et al. 2016; Zhang et al. 2018). Specifically, prolonged dietary insult of male mice resulted in a relative increase of tDR levels in mature spermatocytes. The injection of small RNAs extracted from diet-challenged males into the paternal pronucleus of in vitro–fertilized murine oocytes resulted in measurable and long-lasting effects on metabolic programming in developing offspring (Chen et al. 2016; Sharma et al. 2016; Zhang et al. 2018; Sarker et al. 2019). Notably, the only report addressing the mechanistic details of such reprogramming indicated that specific tDRs acted via a complex signaling cascade impinging on chromatin states, which affected gene expression programs in developing zygotes (Boskovic et al. 2020). Importantly, the microinjection of RNAs into a fertilized oocyte delivered exorbitant amounts of diverse small RNAs; the copy numbers of which can be approximated from the methodological descriptions of these manipulations. Specifically, it was stated that the injected small RNA mass corresponded to about six to 10 times the mass of total RNA that is contained in a single murine spermatocyte (Peng et al. 2012; Chen et al. 2016; Sharma et al. 2016; Conine et al. 2018; Sarker et al. 2019). The RNA content of one mammalian spermatocyte is lower than that of any somatic cell, ranging from 10 to 50 femtograms (fg) in human sperm (Goodrich et al. 2012; Jodar et al. 2013) to any mass between 1 and 100 fg in boar, bull, stallion, mice, or rat (Das et al. 2010; Selvaraju et al. 2017; Zhang et al. 2017; Gòdia et al. 2018; Kasimanickam et al. 2019; Roszkowski and Mansuy 2021). Assuming a mass of 50–100 fg of total RNA per murine spermatocyte, these seminal experiments, which reported on the induction of intergenerational effects by small RNAs, introduced between 0.5 and 1 pg of small RNAs into one male pronucleus in an in vitro–fertilized oocyte. Being agnostic to the actual size distribution of small RNAs contained in a single murine spermatocyte, an RNA mass averaging 750 fg would be equivalent to small RNA copy numbers ranging from 7.805 × 10^7^ (for 18 nt length) to 2.347 × 10^7^ (60 nt length). Likewise, the manipulation of murine embryonic stem cells (mESCs) by lipofection with synthetic tDRs or rsRNAs (200 nM of 30–40 nt) would have exposed 32,000 ESCs to ∼2.247 µg of small RNAs (average copy number of 1.204 × 10^14^) per mL culture medium (Shi et al. 2021). Assuming an RNA transfection efficiency of at least 1%, which is at the lower limit of the observed transfection efficiencies of specific tDRs into HeLa cells (Sanadgol et al. 2022), each mESC would have received ∼3.765 × 10^7^ small RNAs, at a minimum. Of note, the reported average transfection efficiency of 1.7% into HeLa cells, even at the lowest tDR molarity that was tested, would have delivered a threefold tDR excess over the actual copy number produced by severe stress exposure that is required to cause robust tRNA fragmentation (Sanadgol et al. 2022). Importantly, both microinjection and lipofection of cells with these (calculated) RNA copy numbers resulted in measurable changes in gene expression and phenotypic outcomes that included a lasting impact on particular metabolic pathways (Chen et al. 2016; Sharma et al. 2016; Zhang et al. 2018). These back-of-the-envelope calculations to arrive at the RNA copy numbers introduced into cells raise questions about the causation of the reported phenotypes. Were they caused by the impact of specific RNA identities (i.e., tDRs) or mass effects of the RNAs introduced into zygotes or embryonic bodies?

To answer these questions, it is necessary to address several fundamental knowledge gaps. How many RNAs of a particular identity are carried by a single spermatocyte? How many are required to impact specific cellular processes in a biological system such as a fertilized oocyte or developing zygote? How many would be sufficient for such an impact? To answer these questions, the copy numbers of particular small RNA identities contained in a single spermatocyte that has matured under normal, as well as metabolic syndrome–promoting conditions, must be first determined, and then such (physiological) RNA copy numbers should be experimentally tested for their effects on gene expression changes in developing zygotes.

## RESULTS AND DISCUSSION

To determine the copy numbers of various small RNAs in murine spermatocytes, especially of specific tDRs, which had been implicated as active agents in transmitting extra-chromosomal information to the next generation, mobile spermatocytes were collected from the cauda of dissected epididymides from adult mice (aged 3 months up to 1 year), total RNA was extracted from a defined number of spermatocytes, and the mass of total RNA per spermatocyte number was determined. Of note, achieving quantitative RNA extraction from spermatocytes is likely impossible since a considerable proportion of RNAs is embedded in sperm nuclei (Goodrich et al. 2012; Johnson et al. 2015) and therefore might be restricted from efficient release (Goodrich et al. 2012). Hence, the mass of total RNA extracted from spermatocyte preparations is likely lower than expected, which will result in an underestimation of the copy number of specific RNAs per spermatocyte.

**FIGURE 1.**
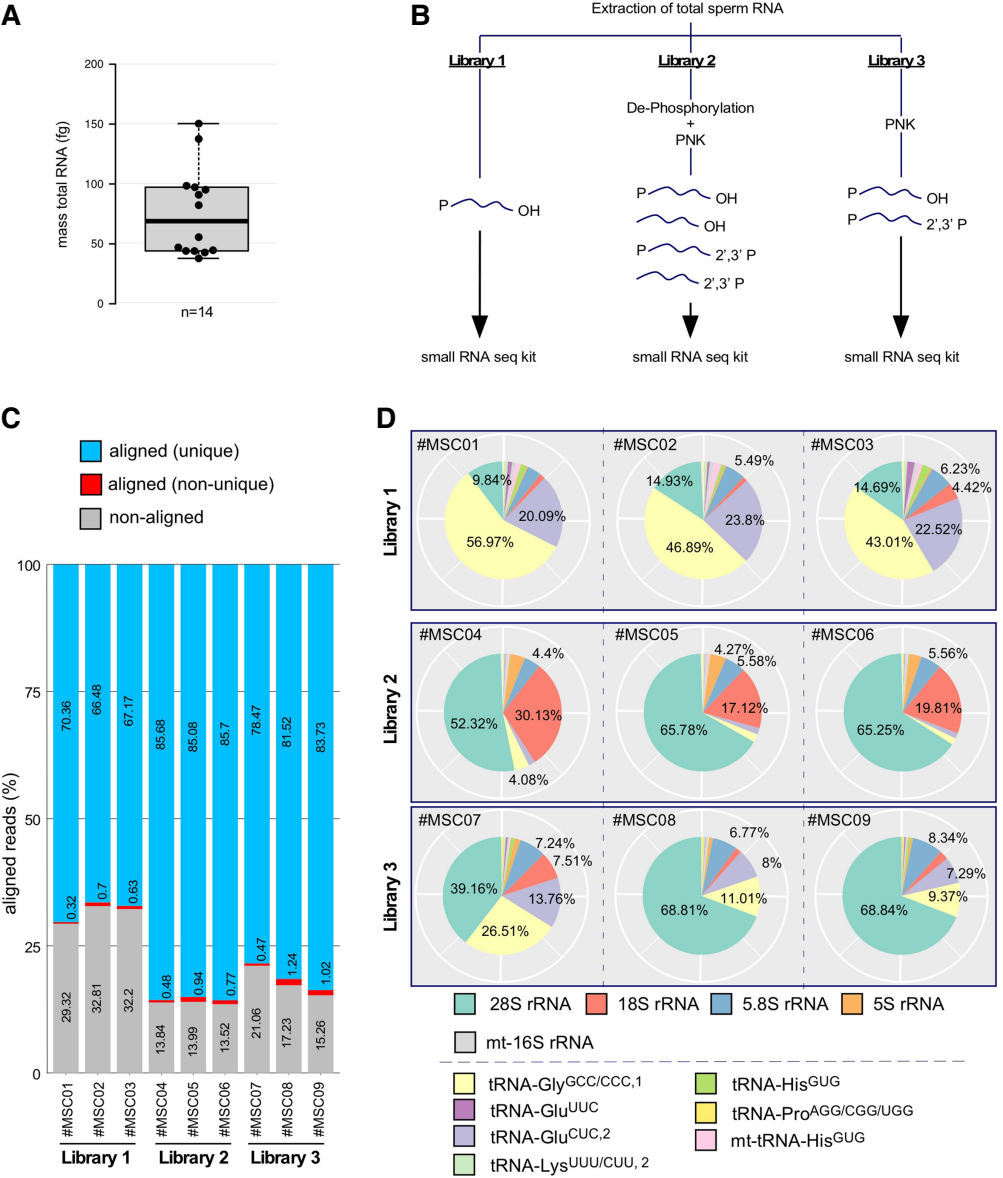
The effects of different library preparations on the read distribution of tDRs and rsRNAs. (*A*) Boxplot summarizing the total RNA content (femtograms, fg) in single murine spermatocytes from the epididymis of mice (*n*=14). (*B*) Cartoon summarizing three different approaches for preparing cDNA from extracted total spermatocyte RNA as input for next-generation small RNA sequencing. (*C*) Statistics of trimmed read alignment (according to cDNA library preparation protocol) to the mouse rRNA/tRNA reference sequence. Aligned reads represent uniquely mapped (blue) and multimapping reads (in red). (*D*) Pie charts showing the relative distribution of reads representing the most abundant RNA species in all sequenced libraries (MSC01–09).

The calculated yield of the total mass that was extractable from preparations of motile murine spermatocytes released from dissected epididymal cauda of male mice (*n* = 14) ranged from 38 to 150 fg RNA per mature spermatocyte (Fig. 1A; Supplemental Table S1). The extracted average mass value of 76 fg RNA per spermatocyte was in line with previous reports quantifying the total RNA content of single murine spermatocytes (Roszkowski and Mansuy 2021). Before attempting to determine absolute copy numbers of individual small RNA identities, their presence in spermatocyte-derived total RNA preparations was confirmed using small RNA sequencing. To do so, cDNA libraries were created using total RNA extracted from the epididymis of pools of males. Library preparation from the same original total RNA pool was performed with three different protocols to compare the relative recovery of small RNAs with different RNA end identities (Fig. 1B). Library 1 (Lib1) was prepared using direct ligation of 3′ and 5′ adapters to sperm RNA. This cDNA library should only include small RNAs and fragments of longer RNAs with preexisting 5′-P residues and 3′-hydroxyl group termini at the time of RNA extraction. Library 2 (Lib2) was prepared after extensive de-phosphorylation (de-P*) of RNA, thereby removing both 5′ and 3′ phosphoryl groups (5′-P, 3′-P) and 3′ cyclic phosphates (3′-cycP), followed by PNK-mediated re-phosphorylation of 5′ ends (P*) and ligation of 3′ and 5′ adapters. This cDNA library should capture all RNA (fragments) independently of their original terminal identities. Library 3 (Lib3) was prepared after selective dephosphorylation of 3′ ends (including cycP) using PNK (without ATP), followed by ligation of 3′ and 5′ adapters. This cDNA library should represent RNAs with preexisting 5′-P and 3′-hydroxyl groups, plus RNA with 3′-P/cycP extremities. cDNA libraries were sequenced in paired-end mode, and mapping was performed to the mouse rRNA/tRNA space (Pichot et al. 2021). These analyses revealed that 66%–71% of reads in Lib1 corresponded to rRNA- and tRNA-derived reads (Fig. 1C). This proportion reached >85% in Lib2 originating from RNA pretreated by de-P*/P*, which indicated that sperm-borne RNAs were overwhelmingly represented by fragments from the most abundant noncoding RNAs, with a majority having 5′-OH and 3′-P/cycP termini (Fig. 1C). Notably, selective de-P* of 3′ ends (Lib3) resulted in a percentage of tRNA- and rRNA-derived reads similar to Lib2, indicating that most RNAs displayed a mix of 5′-P/3′-P (3′-cycP) termini (Fig. 1C).

**FIGURE 2.**
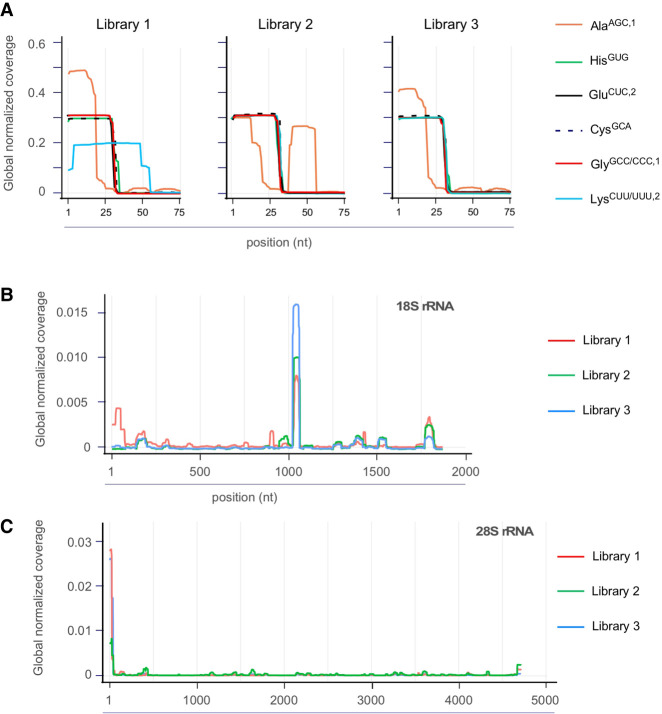
Determining tDR and rsRNA identity in murine spermatocytes. (*A*) Representative overlay metaplots of global normalized coverage for abundant tRNA-derived reads obtained from spermatocyte RNA after using three different cDNA library preparation protocols. The fraction of reads among all tRNA-derived reads is plotted against nucleotide position (1–75) in mature tRNAs containing a 3′ terminal CCA. (*B*) Representative overlay metaplot of global normalized coverage for 18S rRNA-derived reads obtained from (pooled) spermatocyte RNA samples after using three different cDNA library preparation protocols. The fraction of reads among all rRNA-derived reads is plotted against nucleotide position in mature 18S rRNAs. (*C*) Representative overlay metaplot of global normalized coverage for 28S rRNA-derived reads obtained from spermatocyte RNA samples as in (*B*). The fraction of reads among all rRNA-derived reads is plotted against nucleotide position in mature 28S rRNAs.

Separate counting of tRNA- and rRNA-derived reads revealed a substantial difference in library composition when ligation-competent 5′-P/3′-OH termini were compared with total RNA samples (Fig. 1D). As for the identity of sperm-borne tDRs, independently of the cDNA library preparation protocol, the majority was derived from those tRNA isoacceptors, which were previously reported as overrepresented in murine spermatocytes (Peng et al. 2012; Chen et al. 2016; Sharma et al. 2016; Conine et al. 2018). Notably, reads derived from the 5′ moieties of tRNA-Gly^GCC/CCC,1^, tRNA-Glu^CUC,2^, and tRNA-Glu^UUC^, so-called “jackpot” tDRs (Pang et al. 2014), dominated Lib1 (>60%), while reads derived from rRNA species were limited to <20%. In contrast, the percentage of reads from jackpot tDRs was distinctly lower in Lib2 (1.5%–2%), while rRNA-derived reads dominated the libraries (>80%, Fig. 1D). Lib3 revealed a high content of rRNA-derived and an intermediate percentage of reads representing tRNAs (Fig. 1D), confirming rRNA fragments as the major component of sperm-borne RNAs. Inspection of tRNA-derived reads revealed that, independently of the library preparation, jackpot tDRs were represented mostly by 5′ moieties (Fig. 2A). In contrast, other tRNA isoacceptors were represented by reads covering 3′-tRNA halves. Notably, the coverage profiles of long rRNA-derived reads showed an accumulation of reads in specific parts of both 18S and 28S transcripts, unaffected by the cDNA library preparation protocol (Fig. 2B,C).

**FIGURE 3.**
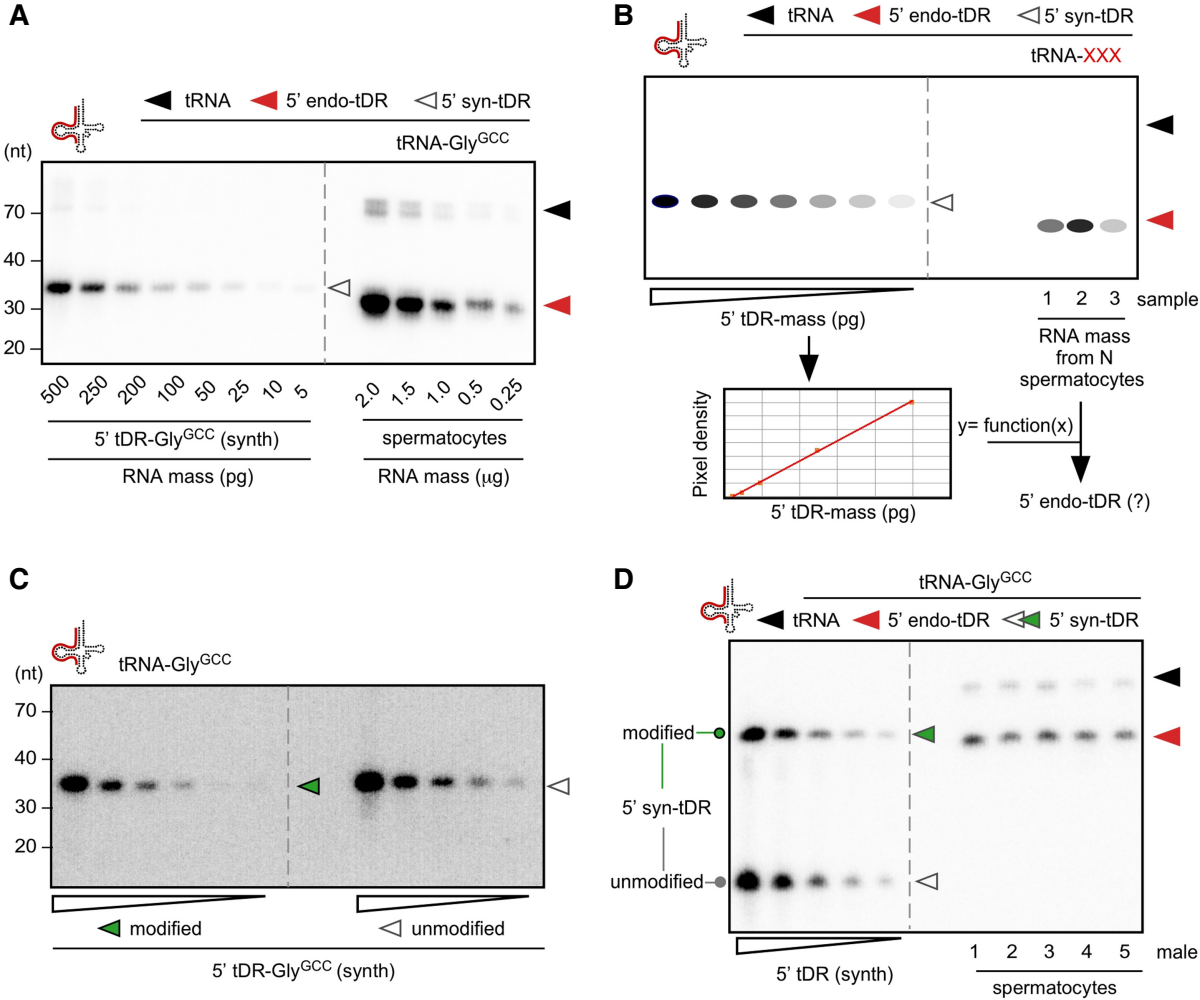
Determining 5′ tDR copy numbers in murine spermatocytes using quantitative northern blotting. (*A*) Northern blotting of a mass titration series of an unmodified 5′ tDR-Gly^GCC^ along with decreasing masses of total RNA corresponding to decreasing numbers of murine spermatocytes using a probe against the 5′ moiety of tRNA-Gly^GCC^. (*B*) Cartoon depicting the approach of quantitative northern blotting on different masses of a synthetic 5′ tDR. Signals obtained by phospho-imaging are used to measure pixel densities, which are then plotted against tDR mass. The derived linear function allows determining a mass value as a function of pixel density recorded from a biological sample that contains 5′ tDRs. (*C*) Representative northern blot of a mass titration series of modified and unmodified 5′ tDR-Gly^GCC^ (500-5 pg) using a probe against the 5′ moiety of tRNA-Gly^GCC^. (*D*) Representative northern blot of a mass titration series of unmodified and modified 5′ tDR-Gly^GCC^ (500-5 pg), loaded sequentially into the same lanes alongside total RNA (50 ng) extracted from epididymal spermatocytes of three different males, using a probe against the 5′ moiety of tRNA-Gly^GCC^. Black arrowheads: mature tRNAs; red arrowheads: 5′ tDRs; empty arrowheads: mass titration series of unmodified RNAs; green arrowheads: mass titration series of modified 5′ tDR-Gly^GCC^.

Northern blotting for tRNA-Gly^GCC^ in total RNA extracted from mature murine spermatocytes confirmed the presence of high levels of “jackpot” 5′ tDR-Gly^GCC^ relative to the parental tRNA-Gly^GCC^ (Fig. 3A). To quantify the copy numbers of 5′ tDR-Gly^GCC^ and other 5′ tDR identities, hybridization-based quantitative northern blotting was performed. To do so, a mass titration series (in the picogram range) of a given synthetic 5′ tDR identity was blotted alongside a known mass of total RNA extracted, representing a defined number of spermatocytes (Fig. 3B). ^32^P-end-labeled DNA probes complementary to the respective tRNA identity were used to collect and record signal intensities per tDR mass standard to derive a linear function of pixel density per tDR mass. Using this function, the mass equivalent of tDRs per mass of total RNA can be converted into actual tDR copy numbers in a given sample (Fig. 3B).

**FIGURE 4.**
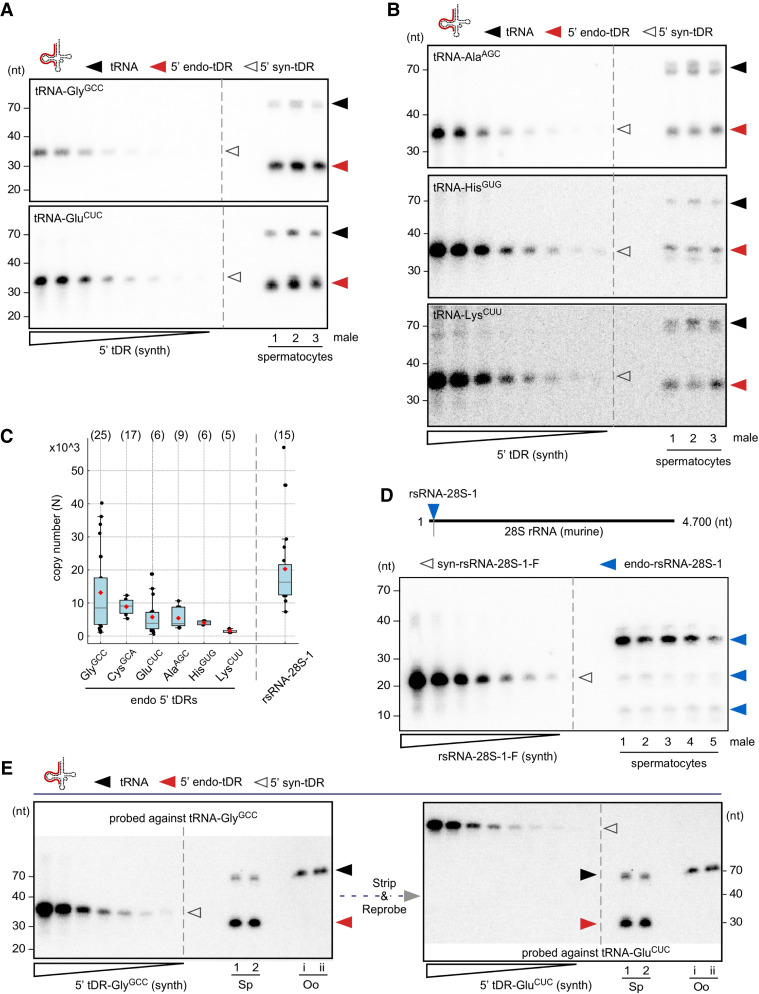
Copy number determination of specific 5′ tDRs in murine spermatocytes and oocytes. (*A*) Representative northern blot of a mass titration series (250-5 pg) of unmodified 5′ tDR-Gly^GCC^ and 5′ tDR-Glu^CUC^ along with total RNA (50 ng) extracted from spermatocytes of mice (*n* = 3) using a probe against the 5′ moiety of tRNA-Gly^GCC^ and 5′ tRNA-Glu^CUC^, respectively. (*B*) Representative northern blot of a mass titration series of unmodified 5′ tDR-Ala^AGC^, -His^GUG^, and -Lys^CUU^ (500-5 pg) alongside total RNA (50 ng) extracted from spermatocytes of mice (*n* = 3) using a probe against the 5′ moieties of the parental tRNAs. (*C*) Boxplots summarizing the calculated copy number of specific 5′ tDRs from different tRNA isoacceptors and one rsRNA (rsRNA-28S-1) per spermatocyte in different individual mice (N above individual boxplots). The median (black horizontal line) and the mean (red diamond) are marked. The individual blots and their analysis, resulting in the displayed data points, are described in the Supplemental Tables S2–S4. (D) Representative northern blotting of a mass titration series of unmodified rsRNA-28S-1 (500-5 pg) alongside total RNA (50 ng) extracted from spermatocytes of mice (*n* = 5) using a probe against the 28S rRNA. (*E*) Northern blotting of total RNA (50 ng) extracted from spermatocytes of mice (Sp, *n* = 2) and total RNA extracted from two pools of germinal vesicle oocytes (Oo, *n* = 100, Roman numerals) alongside a mass titration series of unmodified 5′ tDR-Gly^GCC^ and 5′ tDR-Glu^CUC^ (500-5 pg) using a probe against the 5′ moieties of tRNA-Gly^GCC^ and 5′ tRNAs-Glu^CUC^, respectively. For this blot, two tDR mass titration series were loaded subsequently into the same lanes (unmodified 5′ tDR-Gly^GCC^ followed by unmodified 5′ tDR-Glu^CUC^) along with RNA from biological samples, which was loaded at the time of loading unmodified 5′ tDR-Gly^GCC^. The blot was first probed for tRNA-Gly^GCC^ followed by stripping and re-probing for tRNA-Glu^CUC^. Black arrowheads: mature tRNAs; red arrowheads: 5′ tDRs; empty arrowheads: tDRs or rsRNA-28S-1 mass titration series, respectively; blue arrowheads: rsRNAs-28S-1.

Sperm-borne small RNAs carry various chemical modifications (Chen et al. 2016; Zhang et al. 2018), some of which could affect the hybridization efficiency of complementary DNA probes. To test for the potential effects of chemical modifications on the hybridization efficiency of a DNA probe used to detect tRNA-Gly by quantitative northern blotting, 5′ tDR-Gly^GCC^ was synthesized, containing RNA modifications at positions inferred to be modified in the 5′ moiety of parental tRNA-Gly^GCC^ from human isoacceptors tRNA-Gly^GCC^ and -Gly^CCC^ (Drino et al. 2020). A mass titration series of unmodified and modified 5′ tDR-Gly^GCC^ was probed with the same complementary DNA probe. While ribose-methylated nucleotides (Cm at position 4 in 5′ tDR-Gly^GCC^) and *N*2-methylguanosine (m^2^G at position 6 in 5′ tDR-Gly^GCC^) should not interfere with Watson–Crick base-pairing, dihydrouridine (D at position 20 in 5′ tDR-Gly^GCC^) is known to destabilize duplex structures (Sipa et al. 2007). Accordingly, the quantitative northern blotting results indicated a better hybridization efficiency of the DNA probe toward unmodified 5′ tDR-Gly^GCC^ (by a factor of 1.5–2; Fig. 3D; Supplemental Table S2). This result warrants caution in using synthetic 5′ tDRs for quantitative northern blotting and the calculation of tDR copy numbers in biological samples containing modified tDRs. However, since the calculated factor for 5′ tDR-Gly^GCC^ did not affect the magnitude of the determined tDR copy numbers, together with the impracticability of synthesizing each 5′ tDR identity to be queried with specific modifications, mass titration series of unmodified 5′ tDR identities (tRNA-Gly^GCC^, -Glu^CUC^, -Cys^GCA^, -Ala^AGC^, -His^GUG^, -Lys^CUU^) were probed alongside total RNA from a specific number of mobile murine spermatocytes extracted from different males (Fig. 4A,B; Supplemental Table S3). The obtained hybridization signals were used to calculate the respective tDR mass per sample. Assuming that every spermatocyte contained similar copy numbers of the queried tDR identity, calculated copy numbers of isoacceptor-specific 5′ tDRs ranged from a few thousand to tens of thousands per spermatocyte (Fig. 4C; Supplemental Table S3). Specifically, the calculated average copy number of a specific 5′ tDR identity per spermatocyte was 13,000 molecules for 5′ tDR-Gly^GCC^, 6000–8000 molecules for 5′ tDR-Ala^AGC^ or 5′ tDR-Cys^GCA^, followed by ∼5000 molecules for 5′ tDR-Glu^CUC^ and fewer than 5000 molecules for other isoacceptors such as 5′ tDR-His^GUG^ and 5′ tDR-Lys^CUU^. Factoring in the observed difference in hybridization efficiency based on tDR-Gly^GCC^ would increase these values by at least 1.5-fold.

Of note, ribosomal RNA fragments carried by spermatocytes have been largely ignored by reports focusing on the role of tDRs as causative agents for inducing intergenerational phenotypes in mice. Even though it was known that sperm-borne rsRNAs are degraded products of rRNAs (Johnson et al. 2011; Peng et al. 2012; Chen et al. 2016; Sharma et al. 2016), only very recently has it been acknowledged that rsRNAs could have an impact, too (Shi et al. 2021; Liu et al. 2023). To determine the copy numbers of one prominent rsRNA (rsRNA-28S-1 as reported in Shi et al. 2021), quantitative northern blotting was performed using a mass titration series of synthetic rsRNA-28S-1. The results indicated that rsRNA-28S-1 copy numbers ranged from 7000 to 57,000 molecules with an average of ∼20,000 molecules per spermatocyte extracted from the epididymis cauda of different males (Fig. 4C,D; Supplemental Table S4). These data corroborated the results of our and other small RNA sequencing analyses, which also reported that the relative amounts of rRNA-derived sequences constantly superseded tDR content in male spermatocytes (Shi et al. 2021; Liu et al. 2023).

All reports implicating the degradation products of noncoding RNAs in transferring a still rather ill-defined RNA-coded information from a single spermatocyte into a fertilized oocyte have merely considered that only paternally transmitted small RNAs impact zygotic development. However, RNA sequencing of unfertilized oocytes has revealed the presence of maternally contributed small RNAs, which were composed of repeat-derived RNAs, endo-siRNAs, rsRNAs, piRNAs, miRNAs, and tDRs (Yang et al. 2016). Notably, these published data indicated that the tDR load, relative to all other RNA species present, was very low in an oocyte. However, qRT-PCR suggested that specific tDR identities were relatively more abundant in an oocyte when compared with a single spermatocyte (Yang et al. 2016). Specifically, PCR products derived from 5′ tDR-Glu^CUC^, a jackpot tDR, superseded those amplifiable from single spermatocytes by a factor of ∼3 × 10^5^ (Yang et al. 2016). Using this factor for multiplication with 5 × 10^3^ (the number of 5′ tDR-Glu^CUC^ copies per single spermatocyte, calculated by quantitative northern blotting, Fig. 4C), yielded an estimated 1.5 × 10^9^ copies of 5′ tDR-Glu^CUC^ to be contained in a single unfertilized oocyte. To determine if 5′ tDR-Glu^CUC^ could be detected in oocytes by quantitative northern blotting, total RNA was extracted from pools of 100 germinal vesicle stage murine oocytes (GVOs), which, according to Yang et al. (2016) and our calculations, should contain ∼4.8 × 10^11^ copies of 5′ tDR-Glu^CUC^. Probing a mass titration series of 5′ tDR-Glu^CUC^ alongside total RNA extracted from replicate pools of GVOs (*n* = 2) revealed the presence of parental tRNA-Glu^CUC^ but no 5′ tDR-Glu^CUC^ in GVOs. In contrast, 5′ tDR-Glu^CUC^ signals could be robustly detected in total RNA extracted from 5.0 × 10^5^ spermatocytes (Fig. 4E). Probing GVO-derived total RNA for 5′ tDR-Gly^GCC^, the tDR identity with the highest copy number per spermatocyte, showed similar results as 5′ tDR-Glu^CUC^ (Fig. 4E). These data suggested that jackpot tDR copy numbers are distinctively lower in individual oocytes than reported after amplification-based methods (Yang et al. 2016).

The perceived involvement of particular tDR identities in the inheritance of acquired traits, such as metabolic syndrome from fathers to offspring, has created fertile ground for new concepts postulating pervasive small RNA-mediated reprogramming of zygotic gene expression that mirrors paternal responses to sustained environmental challenge/insult (Zhang et al. 2019; Zhang and Chen 2020; Conine and Rando 2022). After almost a decade of observational studies, it is important to begin moving beyond the descriptive nature of these phenomena and aim to decipher the mechanistic underpinnings of the proposed RNA-mediated reprogramming of gene expression programs. Notably, most conclusions drawn from the original seminal reports and their follow-up studies were based on mere associations or resulted from extreme manipulations of the experimental system, many of which were performed without quantitative insight. Even though the results obtained by the presented quantitative northern blotting approach are likely still an underestimation of actual RNA copy numbers per spermatocyte due to RNA loss during extraction, PAGE, and blotting, the low magnitude of the calculated numbers suggests that a single spermatocyte is unlikely delivering tens of millions but rather (tens of) thousands of a given specific small RNA identity into an oocyte. Notably, all reported changes in sperm-borne tDRs were calculated exclusively based on normalized small RNA reads obtained from spermatocyte-derived cDNA libraries. Even though tRNA and tDR sequencing via cDNA synthesis will likely never become absolutely quantitative (due to biases caused by the impact of RNA structure and modifications), the absolute RNA mass injected into a single oocyte as reported in (Chen et al. 2016; Sharma et al. 2016) can be used to calculate the contribution of specific tDRs within the injected small RNAs. When taking the ∼750 fg of small RNAs (7.805 × 10^7^ mol for 18 nt, plus 2.347 × 10^7^ mol for 60 nt, results in a mean of 5.076 × 10^7^ mol) as basis, and using the relative contribution of “jackpot” tDR reads to the library preparation that revealed their lowest percentage (Lib2, 1.5%–2% as summarized in Fig. 1), one can approximate that the injection of an average of 5.076 × 10^7^ small RNAs (between 18 and 60 nt) contained between 7.614 and 10.152 × 10^5^ mol “jackpot” tDRs. Comparing these calculated values with the values obtained by quantitative northern blotting (13,000 and 5000 mol for 5′ tDR-Gly^GCC^ and 5′ tDR-Glu^CUC^, respectively), one can arrive at an approximate factor of introduced excess (70-fold or 180-fold for tDR-Gly^GCC^ and 5′ tDR-Glu^CUC^, respectively).

Furthermore, the observed difference in the calculated small RNA copy number count from northern blotting between individual males, besides being caused by the handling of extracted RNA, also suggested that the absolute small RNA load per spermatocyte could be variable, even in males raised on a standard diet. This prompts the question whether the reported relative changes in 5′ tDR levels in spermatocytes from males enduring a sustained (>10 weeks) dietary insult that resulted in a max. Fourfold change in specific tDR reads in normalized sequencing libraries (Chen et al. 2016; Sharma et al. 2016) were indeed the cause for the observed metabolic phenotypes in the offspring. To answer this question, it is recommended to adjust the introduction of ectopic small RNAs to much lower copy numbers when manipulating a biological system. Therefore, we suggest that a good starting point for further investigations into RNA-mediated effects during the fertilization process could be to systematically determine the copy number of molecules (RNAs, proteins, cofactors), not only of those that are transferred by one spermatocyte into one oocyte but also of those that are already contained in the oocyte. Having a quantitative idea about the orders of magnitude for copy numbers and putting them into perspective (the oocyte volume is ∼60,000× larger than the incoming sperm head) will be of major importance for developing hypotheses and when designing experiments to address the potential for the regulatory function of small RNAs during zygotic development and beyond.

## MATERIALS AND METHODS

### Sperm collection

Spermatocytes from male mice were collected by dissecting the cauda epididymis (epididymal tail), including the ductus deferens (vas deferens) on both sides. These animals were part of experiments conducted by the Jantsch group at the Center for Anatomy and Cell Biology at the Medical University of Vienna. These experiments were authorized by the Animal Ethics Committee. Reuse of animal tissues in this context represents a reduction in the number of laboratory animals required by the replace, reduce, and refine rules (3Rs). Male mice of various genotypes (Supplemental Table S1) were aged between 11 and 33 weeks. Tissue preparations obtained from individual animals were transferred to a cell culture plate (24 wells) containing 400 µL pre-equilibrated human tubal fluid (HTF) solution and cut into pieces using microsurgical scissors. Plates were incubated for 1 h at 37°C with a 5% CO_2_ atmosphere to allow mobile spermatocytes to leave the tissue. The spermatocyte-containing solution was transferred into an Eppendorf tube. The preparation was centrifuged at 300*g* for 5 min to pellet spermatocytes and white blood cells. The preparation was then left at 37°C for 15 min to allow mobile spermatocytes to swim into the solution. Mobile cells were separated from pelleted and immobile cells and transferred to a new tube for analysis.

### Oocyte collection

All oocytes were collected from hormonally stimulated females (F1 from Bl6 × DBA crosses) at the germinal vesicle (GV) stage as described in Eleftheriou et al. (2022). These animals were part of experiments conducted by the Wossidlo group at the Center for Anatomy and Cell Biology at the Medical University of Vienna. These experiments were authorized by the Animal Ethics Committee. Reusing animal tissues in this context means reducing the number of laboratory animals required following the Replace, Reduce, and Refine Rules (3Rs).

### Cell counting

Extracted sperm in HTF solution was diluted in 1× PBS, placed on ice for at least 20 min to inactivate sperm movement, and stored at 4°C until counting. Spermatocytes were counted using a counting chamber at 400× magnification with a light microscope.

### Total RNA isolation from spermatocytes

The solution containing viable spermatocytes was divided equally into Eppendorf tubes and spun at 500*g* for 5 min. RNA was extracted using 1 mL TRIzol reagent containing 100 mM dithiotreitol (DTT) and incubation at room temperature for 10 min. Samples were added to PhaseMaker tubes, and 200 μL of chloroform was added, followed by mixing and incubation at room temperature for 5 min. Samples were centrifuged at 16,000*g* for 5 min at 4°C. The aqueous phase was precipitated using an equal volume of isopropanol and 1 μL GlycoBlue (Ambion, 15 mg/mL) for ≧15 min at room temperature. RNA was collected by centrifugation at 21,000*g* for 30 min at 4°C. RNA pellets were washed once using 75% ethanol, followed by air-drying for ≦5 min at room temperature. RNA concentration was measured using Qubit (Thermo).

### Total RNA isolation from oocytes

Total RNA from pools of 100 oocytes was extracted using TRIzol and PhaseMaker tubes as described for spermatocyte samples. RNA concentration was measured using Qubit (Thermo).

### Solid phase synthesis of modified RNA

#### Synthesis

The m^2^G and Ѱ phosphoramidites were synthesized in-house (synthetic procedures will be published elsewhere) and were used in combination with commercially available 2′-O-TBDMS protected RNA building blocks (ChemGenes) to synthesize modified 5′ tDR-Gly^GCC^. Controlled pore glass (CPG) RNA solid support (1000 Å pore size, ChemGenes) with an average loading of 40 µmol g^−1^ was used to synthesize the RNAs on an ABI 391 PCR Mate using a self-written RNA synthesis cycle. Amidite (0.1 m) and activator (5-benzylthio-1*H*-tetrazole, 0.25 M) solutions were dried over freshly activated molecular sieves (3 Å) for at least 48 h. The synthesized 5′ tDR-Gly^CCC^ containing pseudouridine (Ѱ) was treated twice with 20 mL of 20% (v/v) diethylamine in acetonitrile and dried under high vacuum before alkaline deprotection.

#### Mild alkaline deprotection

One milliliter methanol and 1 mL ammonia (7 n) in methanol were added to the solid support. The reaction tube was shaken vigorously at room temperature for 72 h. The supernatant was filtered into a 10 mL round-bottom flask. The remaining solid support was washed three times with a mixture of THF/water (1/1), and the liquid phases were combined with the first filtrate and evaporated to dryness. The residual white precipitate was dried in high vacuum for at least 1 h.

#### 2′-O-TBDMS deprotection

After evaporation of the alkaline deprotection solution, the 2′-O-protecting groups were removed by adding 3 mL of 1m TBAF (tetrabutylammonium fluoride) in THF. After 16 h at 37°C, the reaction was quenched by the addition of 3 mL of TEAA buffer pH 7.4 (triethylammonium acetate).

#### Purification and quality check

The solvent was evaporated to ∼1 mL and applied to a HiPrep 26/10 desalting column (GE Healthcare, Austria) using an ÄKTA start system (GE Healthcare). The crude RNA was eluted with water, evaporated to dryness, and redissolved in 1 mL of water. Purification of the RNA sequences was achieved in a single run by applying the crude RNA to a preparative Dionex DNAPac PA-200 column (22 × 250 mm, Eluent A: 25 mM Tris·HCl, 10 mM sodium perchlorate, 20% acetonitrile, pH 8.0; Eluent B: 25 mM Tris·HCl, 600 mM sodium perchlorate, 20% acetonitrile, pH 8.0). The fractions containing the desired target RNAs were pooled and loaded on a C18 Sep-Pak cartridge (Waters) to remove HPLC buffer salts. The RNA sodium salt form was then eluted from the C18 column with water/acetonitrile (1/1, v/v), evaporated to dryness, and transferred to a 1.5 mL reaction tube using 1 mL of water for concentration determination and mass spectrometric analysis. Sample concentrations were determined by measuring UV absorption at 260 nm on a NanoPhotometer (Implen).

#### Mass spectrometry

Purified oligonucleotides were characterized by mass spectrometry on a Finnigan LCQ Advantage MAX ion trap instrument connected to a Thermo Fisher U3000 HPLC in negative-ion mode, with a potential of −4 kV applied to the spray needle. LC: Sample (250 pmol of oligonucleotide dissolved in 20 µL of 20 mM EDTA solution; average injection volume: 10–20 µL); column (Amersham µRPC C2/C18; 2.1 × 100 mm) at 21°C; flow rate: 100 µL min^−1^; eluant A: 8.6 mM TEA, 100 mM 1,1,1,3,3,3-hexafluoro-2-propanol in H_2_O (pH 8.0); eluant B: methanol; gradient: 0%–100% B in A within 30 min; UV detection at 254 nm.f (see Tabel 1).

**TABLE 1.** Quality control of the modified 5′ tDR-GlyGCC

Sequence	Length	Molecular weight		Purity
		Calculated (amu)	Found (amu)	(estimated from HPLC trace)
5′ tDR-Gly^GCC^	32 nt	10,265.11	10,264.38	≥97%

### Northern blotting (NB)

In general, total RNA extracted from spermatocytes or oocytes was separated by 12% urea-PAGE, followed by RNA transfer to nylon membranes (Hybond, GE Healthcare) in 0.5× TBE using a semidry blotting device for 30 min at 10 V = const. Blotted RNA was immobilized by UV-cross-linking with 1200 mJ/cm^2^ (Stratalinker), followed by drying of membranes at 60°C for >12 h. DNA oligonucleotides complementary to tRNA sequences were radiolabeled using T4 PNK and ^32^P-γ-ATP. Unincorporated ^32^P-γ-ATP was removed by desalting using spin columns (P-6DG polyacrylamide, BioRad). Hybridization was performed at 40°C for ≧4 h in a hybridization buffer (5× SSC, 20 mM Na_2_HPO_4_ pH 7.4, 7% SDS, 1× Denhardt's reagent). Membranes were washed once for 10 min with wash buffer A (3× SSC, 5% SDS) at 40°C, followed by wash buffer B (1× SSC, 1% SDS) for 10 min at room temperature. Membranes were exposed to storage phosphor screens (GE Healthcare) and imaged using a Typhoon imager (GE Healthcare).

### Quantification of NB probe efficiency using a specific 5′ tDR

To test if 5′ tDR detection by northern blotting is affected by RNA modifications, a dilution of a defined mass (picogram range) for synthetic 5′ tDR-Gly^GCC^ (tDR-1:32-Gly-GCC-1) sequences (unmodified and modified according to MODOMICS [Cappannini et al. 2023], see oligo sequences) was probed by northern blotting using ^32^P-radiolabeled DNA oligos against the 5′ end of tRNA-Gly^GCC^ (see oligo sequences). Radiographic signals were collected on phosphor-imaging screens (GE Healthcare) and imaged using a Typhoon Scanner (GE Healthcare). Pixel densities from the tDR dilution series were measured and quantified using ImageJ. Pixel densities collected from modified versus unmodified 5′ tDR-Gly^GCC^ were directly compared or plotted against the corresponding tDR mass, and a linear function was generated that allowed for the derivation of a normalized factor that informed on relative probe efficiency.

### Quantification of specific 5′ tDRs and rsRNAs by quantitative northern blotting

For the quantification of 5′ tDRs representing different tRNA isoacceptors, a mass titration series of a synthetic unmodified 5′ tDR sequence was blotted alongside defined RNA masses extracted from mobile spermatocytes of different males or oocyte pools. For some experiments, more than one mass titration series (representing two different tRNA isoacceptors) was queried on the same PA gel alongside the same defined RNA mass extracted from mobile spermatocytes or oocytes. To do so, PA gels were polymerized containing three equal volume layers (15%, 10%, and 8%, bottom to top). A mass titration series representing the first specific 5′ tDR identity was loaded without loading sperm RNA samples. PAGE was performed at 100 V = const. for 10 min, and then 140 V = const. for 45 min, before the second mass titration series representing the second specific 5′ tDR identity was loaded into the same lanes as the first, alongside defined RNA masses extracted from mobile spermatocytes of individual males. PAGE was performed at 100 V = const. for 10 min, followed by 140 V = const. for 30 min, followed by sequential blotting using a probe against one tRNA isoacceptor, followed by stripping of the membrane and blotting with a probe against the other tRNA isoacceptor. Radiographic signals were collected on phosphor-imaging screens (GE Healthcare) and imaged using a Typhoon Scanner (GE Healthcare). Pixel densities from the tDR dilution series were measured and quantified using ImageJ, plotted against the corresponding tDR mass, and a linear function was derived that allowed calculating the mass of the respective 5′ tDR in a given spermatocyte sample. For rsRNA quantification, a mass titration series of a synthetic rsRNA-28S-1 as described by Shi et al. (2021) was used in the same way as 5′ tDRs were. To calculate 5′ tDR (or rsRNA) masses per single spermatocyte (or oocyte), a defined mass of total RNA extracted from a specific number of spermatocytes (or oocytes) was separated using urea-PA gels (12% acrylamide) in parallel with mass dilution series of a specific 5′ tDR (or rsRNA) identity. After northern blotting with specific DNA probes against the respective tRNA (or rRNA) identities, the measured pixel density for tDR or rsRNA signals in a sample was used to calculate the tDR (or rsRNA) mass per total RNA extracted from a given number of cells. The number of tDRs (or rsRNA) per cell was then calculated using the equation: moles ssRNA (mol) = mass of ssRNA (g)/(length of ssRNA [nt] × 321.47 g/mol) + 18.02 g/mol where the nucleotide length (nt) for a small RNA (tDR or rsRNA) reflected the synthetic tDR (or rsRNA) sequence used for quantification. tDR (or rsRNA) copy number was calculated as moles of ssRNA × 6.022 × 10^23^ molecules/mol.

### Small RNA sequencing

Total RNAs from spermatocytes of different males (with different genotypes) were extracted separately (Supplemental Table S2). For cDNA synthesis, RNAs from different males were pooled before cDNA synthesis: pool 1 (males #1 and #6); pool 2 (males #2 and #8); and pool 3 (males #3 and #10). Each pooled sample was split into three, and each sample was subjected in parallel to three different cDNA library preparation protocols (MSC01–MSC09). MSC01–03, corresponding to small RNA pools 1, 2, and 3, respectively, were directly converted to a library using the NEBNext Small RNA Library Prep Set for Illumina (NEB E7330S, USA), following the manufacturer's recommendations. MSC04–06, corresponding to small RNA pools 1, 2, and 3 small RNAs, respectively, were end-repaired. To this end, RNAs were dephosphorylated using Antarctic phosphatase (NEB ref M0289L) for 30 min at 37°C, followed by phosphatase inactivation for 5 min at 65°C. RNAs were directly phosphorylated using T4 PNK (NEB ref M0201L) in the presence of 1 mM ATP for 1 h at 37°C, followed by purification using the RNeasy MinElute Cleanup kit according to the manufacturer's recommendations, except using 675 µL 96% ethanol for the RNA-binding step. Elution of purified RNA fragments was performed in 9 µL of nuclease-free water. RNA fragments were converted to a library using the NEBNext Small RNA Library Prep Set for Illumina (NEB, E7330S), following the manufacturer's recommendations. MSC07–09, corresponding to small RNA pools 1, 2, and 3, respectively, were 3′-end dephosphorylated using 10 U of T4 PNK in 200 mM Tris-HCl (pH 6.5), 200 mM MgOAc, 10 mM β-mercaptoethanol for 6 h at 37°C, followed by PNK inactivation for 20 min at 65°C. Small RNAs were then purified using the RNeasy MinElute Cleanup kit according to the manufacturer's recommendations, except using 675 µL 96% ethanol for the RNA-binding step. Elution of purified RNA fragments was performed in 9 µL of nuclease-free water. RNA fragments were converted to a library using the NEBNext Small RNA Library Prep Set for Illumina (NEB E7330S), following the manufacturer's recommendations. DNA libraries were quantified using a fluorometer (Qubit 2.0 fluorometer, Invitrogen, USA) and qualified using a High-Sensitivity DNA chip on an Agilent Bioanalyzer 2100. Libraries were multiplexed and subjected to high-throughput sequencing on an Illumina NextSeq 2000 instrument in a 2 × 50 bp paired-end (PE) mode.

### Analysis of small RNA sequencing data

Paired-end raw sequencing reads were trimmed by trimmomatic v0.39 in palindrome adapter recognition mode to remove adapter sequences. Trimmed Read1 and Read2 pairs were used for bowtie2 v2.4.2 alignment in paired-end mode. Only concordantly aligned pairs were used for further analysis. Coverage for tRNAs and rRNAs was calculated using SAMtools mpileup utility and using the reduced *Mus musculus* rRNA/tRNA reference sequence containing cytoplasmic and mitochondrial rRNAs, as well as 44 cytoplasmic and 22 mitochondrial tRNAs. Ambiguously aligned sequencing reads were excluded from further analysis.

### Oligonucleotide sequences (5′-3′)

#### Synthetic RNA sequences

5′ tDR-Gly^GCC^ (unmodified): GCA UUG GUG GUU CAG UGG UAG AAU UCU CGC CU
5′ tDR-Gly^GCC^ (modified): GCA JUL GUG GUU CAG UGG DAG AAU UCU CGC CU
(J4: Um; L6: m2G; D19: D)
5′ tDR-Glu^CUC^ (unmodified): UCC CUG GUG GUC UAG UGG UUA GGA UUC GGC GCU C
5′ tDR-Ala^AGC^ (unmodified): GGG GGU GUA GCU CAG UGG UAG AGC GCG UGC
5′ tDR-Cys^GCA^ (unmodified): GGG GGU AUA GCU CAG GGG UAG AGC AUU UGA CU
5′ tDR-His^GUG^ (unmodified): GCC GUG AUC GUA UAG UGG UUA GUA CUC UGC GUU
5′ tDR-Lys^CUU^ (unmodified): GCC UGG AUA GCU CAG UCG GUA GAG CAU CAG ACU
rsRNA-28S-1 (unmodified): AGA CGU GGC GAC CCG CUG AAU UU

#### Northern blotting

According to https://gtrnadb.ucsc.edu/genomes/eukaryota/Mmusc39/mm39-tRNAs.fa

5′ tRNA-Gly^GCC^: TCT ACC ACT GAA CCA CCA AT
detects Mus_musculus_tRNA-Gly^GCC^-2-1 to 2-8, tRNA-Gly^GCC^-3-1, and tRNA-Gly^GCC^-4-1
5′ tRNA-Glu^CUC^: GAA TCC TAA CCA CTA GAC CAC
detects Mus_musculus_tRNA-Glu^CUC^-1-1 to 1-9
5′ tRNA-Ala^AGC^: GCA CGC GCT CTA CCA CTG AGC TAC ACC CCC
detects Mus_musculus_tRNA-Ala^AGC^-1-1 and tRNA-Ala^AGC^-2-1 and -2-2
5′ tRNA-Cys^GCA^: CAA ATG CTC TAC CAC TGA GCT ATA CCC CC
detects Mus_musculus_tRNA-Cys^GCA^ -1-1 and -1-2
5′ tRNA-His^GUG^: CGC AGA GTA CTA ACC ACT ATA CGA TCA CG
detects Mus_musculus_tRNA-His^GUG^-2-1 to 2-8
5′ tRNA-Lys^CUU^: TCT CAT GCT CTA CCG ACT GAG CTA GCC GG
detects Mus_musculus_tRNA-Lys^CUU^-3-1 to 3-7
rsRNA-28S-1: ATT CAG CGG GTC GCC ACG TCT

## DATA DEPOSITION

Small RNA sequencing data have been deposited at ENA (https://www.ebi.ac.uk/ena/) under the accession number PRJEB85978.

## SUPPLEMENTAL MATERIAL

Supplemental material is available for this article.
